# Internally Controlled, Generic Real-Time PCR for Quantification and Multiplex Real-Time PCR with Serotype-Specific Probes for Serotyping of Dengue Virus Infections

**DOI:** 10.1155/2011/514681

**Published:** 2011-12-05

**Authors:** Sandra Menting, Khoa T. D. Thai, Tran T. T. Nga, Hoang L. Phuong, Paul Klatser, Katja C. Wolthers, Tran Q. Binh, Peter J. de Vries, Marcel Beld

**Affiliations:** ^1^Royal Tropical Institute, KIT Biomedical Research, Meibergdreef 39, 1105 AZ Amsterdam, The Netherlands; ^2^Division of Infectious Diseases, Academic Medical Center, Tropical Medicine & AIDS, Meibergdreef 9, 1105 AZ Amsterdam, The Netherlands; ^3^Center for Infection and Immunity (CINIMA), Academic Medical Center, University of Amsterdam, 1105 AZ Amsterdam, The Netherlands; ^4^Division of Microbiology, Cho Ray Hospital, 201B Nguyen Chi Thanh Street, District 5, Ho Chi Minh City, Vietnam; ^5^Division of Medical Microbiology, Academic Medical Center, Section of Clinical Virology, Meibergdreef 9, 1105 AZ Amsterdam, The Netherlands

## Abstract

Dengue has become a global public health problem and a sensitive diagnostic test for early phase detection can be life saving. An internally controlled, generic real-time PCR was developed and validated by testing serial dilutions of a DENV positive control RNA in the presence of a fixed amount of IC with results showing a good linearity (*R* ^2^ = 0.9967) and a LOD of at least 1.95 × 10^4^ copies/mL. Application of the generic PCR on 136 patient samples revealed a sensitivity of 95.8% and specificity of 100%. A newly developed multiplex real-time PCR with serotype-specific probes allowed the serotyping of DENV for 80 out of 92 (87%) generic real-time PCR positive patients. Combined these real-time PCRs offer a convenient diagnostic tool for the sensitive and specific quantification of DENV in clinical specimens with the possibility for serotyping.

## 1. Introduction

Mosquito-borne flavivirus infections such as dengue have rapidly spread and are the most significant and dreaded infectious diseases in the world, in terms of morbidity and mortality [1, 2]. Recent estimates indicate that over 3.5 billion people (*∼*55%) of the world population live in areas at risk for dengue [3]. Worldwide there are 50–100 million cases of dengue infections per year, which result in 25,000 deaths. Dengue has become a major international public health problem due to the expanding geographic distribution of the vector in tropical and subtropical countries [1]. Increased international travel accompanied with increasing transmission or re-emergence and changing epidemiology of dengue in various (sub) tropical countries contribute to a steady rise in confirmed dengue among ill-returned travelers [4]. Dengue is caused by an RNA virus (DENV). DENV is primarily transmitted through bites of the infected *Aedes aegypti* mosquito vector. The majority of DENV infections, with any of the four different virus serotypes (DENV-1, DENV-2, DENV-3, and DENV-4), are mildly asymptomatic and often difficult to recognize in the early phase of infection because signs and symptoms are nonspecific and resemble other febrile illnesses. Only a small number of DENV infections (~5%) will result in severe forms of the disease [5, 6]. The most used diagnostic tools for confirmation of DENV infections are based on detection of antibodies (Ab) or, recently, NS1 antigen (Ag) detection [7]. However, both Ab and Ag detection methods do not distinguish the respective DENV serotypes. Tests based on detection and serotyping of dengue virus offer the possibility to look better at the association between complications of the life threatening DHF and DSS, serotype, and sequential infections [8, 9]. Other real-time PCR assays for DENV infections are based upon type-specific primers but all without internal control (IC) [8, 10, 11]. For reliable detection and to rule out false negative results the use of an IC is essential [12]. Serotyping of DENV is important because the lack of cross-reactive immunity for the four different DENV serotypes may lead to a life threatening complication [9]. In this study we developed and validated an internally controlled generic real-time PCR for detection and quantification of all known DENV serotypes and a multiplex PCR for discrimination of serotypes based upon serotype-specific probes.

## 2. Materials and Methods

### 2.1. Primers and Probes

All known complete genome sequences of DENV found in GenBank were aligned using Vector NTI Advance version 10 and ClustalW. The generic primer pair used for amplification of DENV and of DENV-1 *in vitro *RNA for use as positive control (DENV *in vitro* RNA) is located in the 3′-UTR region. The DENV generic primers, the primers of the noncompetitive internal control RNA (IC) (MS2 RNA; Roche Diagnostics), and the probes sequences for amplification and detection are listed in Table 1. The specific MS2 RNA primers, probe, and characteristics were designed by us. All primers, probes, and linkers were obtained from Biolegio (Biolegio, Nijmegen, The Netherlands).

### 2.2. Construction of the DENV-1 *In Vitro* RNA Positive Control (DENV In Vitro RNA)

For the construction of DENV* in vitro* RNA two linkers were designed, named DENV-hyb-1 and DENV-hyb-2. The sequence of DENV-hyb-1 is (DENV generic primers sequences are in Italic) 5′-*GGTTAGAGGAGACCCCTCCC*AAGTCACAACGCAGCAGCGGGGCCCAACACCAGGGGAAGCTGTACCCTGGTGGTAAGGACTAGAGGTTAGAGGAGACCCCCCGCGCAACAATAAACAGCATATTGACGCTGGGAGAGACCAGAGATCCTGCTGTCTC-3′ and of DENV-hyb-2 is 5′-*GAGACAGCAGGATCTCTGGTCTCTCCC*AGCGTCAATATGCTGTTTATTGTTGCGCGGGGGGTCTCCTCTAACCTCTAGTCCTTACCACCAGGGTACAGCTTCCCCTGGTGTTGGGCCCCGCTGCTGCGTTGTGACTTGGGAGGGGTCTCCTCTAACC-3′. DENV *in vitro* RNA was constructed as described earlier [12, 13].

### 2.3. RNA Quantification

The DENV *in vitro* RNA and MS2 RNA were quantified by measuring the optical density at 260 nm. Serial dilutions of the RNAs were stored at −70°C until use.

### 2.4. Nucleic Acid Extraction, cDNA, and Generic Real-Time PCR

DENV *in vitro* RNA and the 136 patient samples were extracted in the presence of a fixed amount of IC and cDNA was made. The IC was spiked before extraction into lysis buffer containing the sample. The extraction of nucleic acids (NA) was performed on 200 *μ*L serum or plasma sample and 5 *μ*L IC and eluted in 100 *μ*L TE-buffer [14]. 80 *μ*L of the NA extract was used for cDNA synthesis (RT-reaction) as earlier described [12] using random primers (Roche Diagnostics) and the cDNA products were stored at −20°C. For the preparation of cDNA of DENV *in vitro* RNA the DENV *in vitro* RNA and IC were spiked into the lysis buffer containing DENV negative human serum before extraction. The generic PCR mix contained 12.5 *μ*L of 2x Probes Master Mix (Roche Diagnostics), 500 nM of primer (each), 300 nM of DENV-generic-MGB probe and 100 nM of MS2 probe, and 10 *μ*L of cDNA (corresponding to 1/12.5 of extraction) in a 25 *μ*L volume. PCR reactions were performed in Bio-Rad iQ5 real-time machine (Bio-Rad), as followed: 10 minutes at 50°C and 10 minutes at 95°C, followed by 45 cycles of 20 s at 95°C, and 1 minute at 60°C for annealing and extension. Data were analyzed using the Bio-Rad iQ5 software version 2.1 (Bio-Rad). A patient sample was considered positive in the generic PCR if the Cq value was below 40. The negative results were truly negative if the IC was detected in the samples. Otherwise the sample was considered to be unsuitable for analysis and the PCR was repeated for a new extraction.

### 2.5. Serology

Antibodies against DENV were measured using DENV IgM and IgG Capture ELISA (PANBIO) and by the method earlier described by Tran et al. [15].

### 2.6. Clinical Samples

Samples (serum or plasma) from 109 patients with an antibody positive dengue serology and 27 with an antibody negative dengue serology were included. The 109 patients with an anti-DENV positive serology stratified in 26 patients with a primary DENV infection and 83 patients with a secondary DENV infection. The 136 patients could be further divided into 3 groups: 46 ill-returned travelers (21 primary infections, 16 secondary infections, and 9 antibodies negative) [16], 72 patients from Vietnam (5 primary infections and 67 secondary infections), [17] and 18 patients from whom 16 patients were antibody and PCR positive for hepatitis C (HCV) and 2 patients were antibody and PCR positive for hepatitis A (HAV). The 118 of 136 patients (46 ill returned travellers and 72 patients from Vietnam) were tested by our generic PCR and previous by the earlier described type-specific primer PCR [11]. The 18 patients with HCV or HAV were tested only in our generic PCR.

### 2.7. Serotyping by SYBR-Green

The SYBR Green reactions were performed in the Bio-Rad iQ5 real-time machine (Bio-Rad) using the cDNA (10 *μ*L) in a 25 *μ*L volume consisting of 12.5 *μ*L of iQ SYBR Green Supermix (Bio-Rad), 500 nM generic DENV primer pair. For amplification the following PCR program was used: 10 minutes at 95°C, followed by 45 cycles of 20 s at 95°C, and 1 minute at 60°C for annealing and extension. The melting temperature (*T*_*m*_) curve analysis was performed following the amplification and included one cycle of denaturation at 95°C for 1 minute, followed by 60°C for 1 minute and a ramp to 95°C at a rate of 0.1°C with continuous fluorescence measurement, using the iQ5 real-time PCR machine, and the data were analyzed using Bio-Rad software version 2.1 (Bio-Rad).

### 2.8. Serotyping in a Multiplex Real-Time PCR Assay

Serotypes were determined using the generic DENV primer pair and 4 different serotype-specific DENV-probes in one multiplex PCR reaction. Multiplex PCR reactions were performed as described for the generic PCR with 500 nM of DENV primer (each) and 300 nM of each serotype-specific DENV-probe 1–4 (Table 1).

### 2.9. Statistical Analysis

Statistical analyses were performed using SPSS software for windows (version 16.0). Frequencies, means, or medians were calculated to describe background variables. Agreement was expressed by Cohen's kappa value. The type-specific primer PCR by Laue et al. was used as gold standard for calculation of sensitivity and specificity of our assays [11].

## 3. Results

### 3.1. Determination of the Dynamic Range of the Internally Controlled, Generic Real-Time PCR for DENV

For reliable detection and to rule out false negative results an IC was used throughout the whole process of extraction, cDNA synthesis, and PCR reaction. To determine the dynamic range of the internal control the extraction, cDNA synthesis, and PCR procedure were performed on serial dilutions of IC spiked into the lysis buffer containing negative serum. Limiting dilution results for the IC revealed a detection limit of 10 copies/PCR with a 100% hit rate (Table 2). However, for reliable detection of the IC in a duplex assay an input corresponding to 500 copies IC/PCR was chosen which had no influence on the detection limit of DENV *in vitro *RNA (results not shown). In subsequent experiments a fixed amount of 6.25 × 10^3^ IC copies was spiked in the lysis buffer prior to extraction and cDNA synthesis. The IC showed good stable Cq-values (mean Cq-value of 33.34) during the validation.

**Figure 1:**
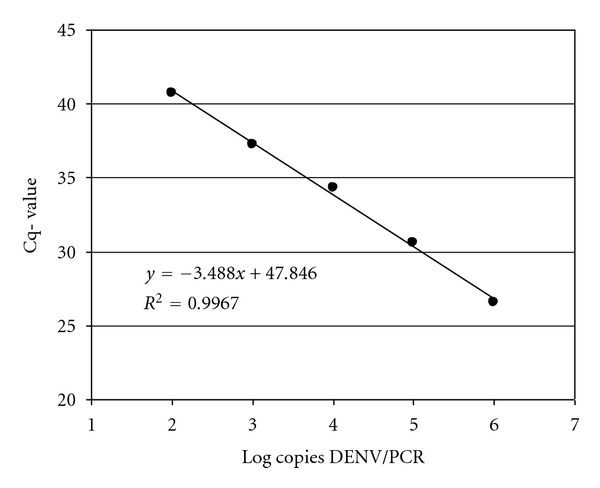
The dynamic range of generic of PCR. Mean Cq-values of twelve replicates per each 10-fold dilutions of DENV* in vitro* RNA extracted with a fixed amount of 6.25 × 10^3^ IC copies in extraction in the back ground of DENV negative serum and tested in generic real-time PCR resulting in a dynamic range of 6.25 × 10^3^–6.25 × 10^7^ copies/mL of DENV* in vitro* RNA with a regression coefficient of 0.9967.

To determine the dynamic range of the generic PCR, we spiked 10-fold serial dilutions of DENV* in vitro* RNA together with a fixed amount of IC into lysis buffer containing DENV negative serum prior to extraction and cDNA synthesis. Subsequently 10 *μ*L of the cDNA was used in PCR. Limiting dilution of DENV* in vitro* RNA resulted in a quantitative detection limit of 10^3^ copies/PCR with a 66.7% hit-rate (Table 3) and a regression coefficient of 0.9967 with a dynamic range of 6.25 × 10^3^–6.25 × 10^7^ copies/mL (Figure 1).

### 3.2. Determination of the Lower Limit of Detection (LOD)

To determine the LOD of the generic PCR assay 2-fold serial dilutions of DENV* in vitro* RNA were performed with 20 replicates per dilution with fixed amount of IC in the background of DENV negative human serum each separately spiked in the lysis buffer prior to extraction. The LOD for DENV* in vitro* RNA was at least 312 copies/PCR corresponding to 1.95 × 10^4^ copies/mL (Table 4).

### 3.3. Intra- and Inter-Assay Variation

The intra- and inter-assay variation was measured in four replicates per dilution of 10-fold dilution series with fixed amount of IC in a background of DENV negative serum: For intra-assay variation determined in three runs performed at one day (results not shown) and for inter-assay variation performed in three 3 runs over 3 consecutive days. The assay variation showed stable Cq-values for the IC and each serial dilution of DENV *in vitro* RNA (Table 5). 

### 3.4. Testing of Clinical Samples

A fixed amount of IC (5 *μ*L) and 200 *μ*L of the patient sample were added to the lysis buffer prior to extraction and cDNA synthesis. Overall, the generic PCR showed a sensitivity of 95.8% and a specificity of 100% (Table 6). All patient samples with detected IC and DENV negative were truly negative. Just four patient samples that tested positive in the type-specific primer PCR tested negative in the generic PCR. The patient samples had been tested earlier by the type-specific primer PCR [16, 17] and the extra freeze-thaw steps may have affected the quality of the RNA in these four samples that showed already a high Cq-value close to 37 or higher in the type-specific primer PCR. A lower percentage (62.2%) of the anti-DENV positive ill-returned travelers had a positive test result in the generic PCR compared with the anti-DENV positive Vietnamese patients (95.8%) which correlates with the longer duration of illness in the ill-returned travelers before seeing a clinician (Table 6).

### 3.5. Serotyping by SYBR-Green

Since the SYBR-green real-time PCR requires no type-specific primers or probes, results can be obtained with one real-time PCR program for the detection of the specific melting curve (*T*_*m*_). We used the cDNA from a panel of patients which were known to be infected with DENV serotype 1 to 4 and who tested previously positive in our generic PCR. The *T*_*m*_ curves however for DENV-1, DENV-2, DENV-3, and DENV 4 were too close to each other (84.90°C, 83.60°C, 85.80°C, and 84.70°C) to enable discrimination between all four serotypes. Also, differences in the viral loads influenced melting temperatures make serotyping of DENV by SYBR-green PCR unreliable (results not shown).

### 3.6. Serotyping by Multiplex Real-Time PCR with Serotype-Specific Probes

The multiplex PCR with serotype-specific probes was only applied on the cDNAs from the 92 patient samples which had a positive test result in the generic PCR. The DENV serotype from these samples was previously determined using the type-specific primer PCR (Table 7) and was tested blindly in our multiplex PCR. The generic PCR detected all 4 DENV serotypes. The multiplex PCR with the type-specific probes correctly identified the DENV serotype in 80 out of the 92 (87%) patient samples. These 12 patient samples which could not be serotyped in our multiplex assay had Cq-values close to 38 or higher in the generic PCR. In three patient samples an infection by two serotypes was detected with the type-specific primer PCR, whereas by the multiplex PCR with serotype-specific probes an infection by two serotypes was detected in seven out of the 80 patient samples (Table 7). The viremia levels in first serotype were higher than in the second serotype.

## 4. Discussion

DENV infections have become a major international public health problem due to the increasing geographic distribution of the vector [1], the increased international travel accompanied with increasing confirmed DENV infection among ill-returned travelers [4], and the lack of cross-reactive immunity for the four different DENV serotypes and hyperendemic circulation of the four serotypes in the same regions. These all are factors that play a significant role in the threat posed by DENV. 

**Figure 2:**
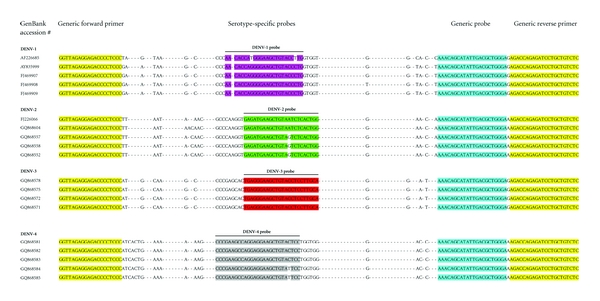
Multiple sequence alignments of DENV 3′UTR region deduced from complete genome sequences of dengue serotypes 1–4 (DENV 1–4) obtained from GenBank showing primers and probes sequences used in generic and/or multiplex real-time PCR with serotype-specific probes for detection of DENV *in vitro *RNA and DENV in patient samples.

An inherent problem in real-time PCR is the presence of amplification inhibitors which may cause false-negative results. MS2 RNA is an ideal “noncompetitive” internal control for use as a process control which have already been proven by other studies. MS2 RNA is commercially available for any user, and the RNA is a stable, noninfectious and absent from human clinical samples, and is a noncompetitive control which does not react with the selected primers and probe for DENV. Our generic real-time PCR assay was capable to amplify the four known serotypes in one PCR reaction and showed a good linearity (*R* ^2^ = 0.9967) with a dynamic range of 6.25 × 10^3^–6.25 × 10^7^ copies/mL and a LOD of at least 1.95 × 10^4^ copies/mL. The intra- and inter-assay variation showed stable Cq-values (SD < 0.70) for each 10-fold serial dilution for DENV* in vitro* RNA. A viral load of 6.25 × 10^3^ copies/mL can still be detected with certainty at a Cq-value of 40 in our generic PCR in 8% of the cases (Table 3). Therefore, a patient sample was considered positive in the generic PCR if the Cq-value was below 40. The DENV generic primers and the various probes are based on a highly conserved region in the 3′-UTR of DENV (Figure 2). DENV genetic diversity and quasi-species could contribute to PCR accuracy as has been shown for HCV, another member of the family of Flaviviruses. However, the variation and the proportion of biological variants are very low in DENV (unpublished data). The target of the described DENV PCR is very small (157 bp) and we cannot describe quasi-species—like in, for example, HCV, and moreover there is no external selection pressure like DENV-specific antivirals or a vaccine affecting the sequence of the amplicon we used for detection of DENV.

 The comparison between the described generic real-time PCR and the earlier described type-specific primer PCR using 118 patient samples showed an excellent concordance between the two assays with a sensitivity of 95.8% and specificity of 100% [11]. The patient samples that were not detected in our generic PCR were quantified with a very low viral load (about 4.16 × 10^4^ copies/mL) in the type-specific primer PCR. The extra freeze-thaw steps caused by using the same sample tube for serological tests, for the type-specific primer PCR assay, and for our generic PCR assay may lead to degradation of DENV RNA by RNases in the sample. Patient samples may be divided into aliquots to minimize the effect of freeze-thawing.

We tried the SYBR-green real-time PCR for serotyping because it is less expensive and easier to perform compared to type-specific primers or probes assays. The SYBR-green assay results showed that both the viral load and the close similarity between the four serotypes influenced the melting temperatures of each serotype (data not shown), making it difficult to obtain reliable results, which were also seen in previous studies [10, 18]. Therefore, we developed a multiplex real-time PCR assay using four serotype-specific probes in one PCR reaction which showed reliable results to discriminate between the 4 DENV serotypes in patient samples. We were able to differentiate between the serotypes in 80 out of 92 patient samples, which had positive generic PCR result, with the multiplex assay (sensitivity of 87%), and 7 coinfections with two serotypes were found. The 12 patient samples which could not be serotyped had Cq-values close to 38 or higher in the generic PCR which was probably due to the differences in sensitivity between the generic and multiplex PCR and competition within the multiplex PCR. A total of 109 patients had positive antibodies against DENV and 92 patients were positive in our generic PCR. This difference may be explained by the relatively high median days of ill-returned travelers after onset of symptoms before visiting a clinician and multiple freeze-thaw steps. The ill-returned travelers which had a negative PCR result but positive antibodies against DENV visited a clinician after a median of 6 days (primary infection; range 1 to 12 days) and 8.5 days (secondary infection; range 4 to 22 days) of illness and possibly the antibodies had emerged after DENV had already disappeared from the blood. It has been postulated that DENV can be detected approximately until 5 days after onset of symptoms by PCR, regardless of the serotype [11].

Our study describes a specific, sensitive, and internally controlled generic real-time PCR assay for early detection of DENV and the possibility for serotyping by multiplexing. Screening patients with the internally controlled, generic PCR assay for DENV and applying the multiplex PCR assay for typing only on positive sample will be more cost-effective than performing a typing PCR on all samples. Many of the published multiplex assays use mixtures of serotype type-specific primers and probes which makes the assay less sensitive and more expensive [10]. Other multiplex PCRs are not genuine multiplex PCR assays because they consist of two separate duplex PCR assays, each with a probe pair for DENV 1–3 or DENV 2–4 [18].

The described assays are an important complementary tool for diagnosis of DENV in patients in the early phase of disease.

## Figures and Tables

**Table 1:** Primers and fluorescence-labeled probes of DENV and noncompetitive IC (MS2 RNA).

Primer or probe	Sequence (5′–3′)	5′ reporter	3′ quencher
DENV-F (10546)-generic	GGTTAGAGGAGACCCCTCCC		
DENV-R (10711)-generic	GAGACAGCAGGATCTCTGGTCT		
MS2-F	CATAAGTTAGATGGCCGTCTGT		
MS2-R	TAGAGACGACAACCATGCCAAAC		
DENV-generic-MGB probe	AAACAGCATATTGACGCTGGGA	FAM	BHQ1
MS2 probe	TCCAGACAACGTGCAACATATCGCGACGTATCGTGATATGG	HEX	BHQ1
DENV-1-MGB probe	AACACCATGGGAAGCTGTACCCTG	FAM	BHQ1
DENV-2-MGB probe	GAGATGAAGCTGTAGTCTCACTGG	HEX	BHQ1
DENV-3-MGB probe	TGAGGGAAGCTGTACCTCCTTGCA	Texas Red	BHQ2
DENV-4-MGB probe	CCCGAAGCCAGGAGGAAGCTGTACTCC	CY5	BHQ2

Nucleotide numbering for generic primers DENV-F and -R is relative to DENV-1 (reference strain AF226685).

F: forward primer; R: reverse primer; MGB: minor groove binding; BHQ1 or 2: black hole quencher 1 or 2.

**Table 2:** Dynamic range of IC^a^.

IC copies in extraction	IC copies in RT	IC copies in PCR^b^	Number positives IC (%; mean Cq-value & SD)
1.25 × 10^7^	10^7^	10^6^	4/4 (100%; 20.74; 0.23)
1.25 × 10^6^	10^6^	10^5^	4/4 (100%; 24.80; 0.43)
1.25 × 10^5^	10^5^	10^4^	4/4 (100%; 27.92; 0.48)
1.25 × 10^4^	10^4^	10^3^	4/4 (100%; 32.08; 0.71)
1.25 × 10^3^	10^3^	10^2^	4/4 (100%; 34.53; 0.56)
1.25 × 10^2^	10^2^	10^1^	4/4 (100%; 36.93; 0.72)
0	0	0	0/4

^a^The dynamic range was determined on 10-fold serial dilution of IC spiked into lysis buffer containing DENV negative human serum as background with 4 replicates for each dilution.

^b^Number of copies cDNA/PCR has been calculated as 1/12.5 of the extracted RNA, assuming 100% efficiency in extraction, reverse transcription (RT), and PCR.

SD: standard deviation.

**Table 3:** Dynamic range of generic real-time PCR^a^.

DENV *in vitro* RNA copies in extraction	DENV *in vitro* RNA copies in RT	DENV *in vitro* RNA copies in PCR^b^	IC copies in PCR^b^	Number positives DENV* in vitro *RNA (%; mean Cq-value: SD)	Number positives IC (%; mean Cq-value: S.D)
1.25 × 10^7^	10^7^	10^6^	500	12/12 (100%; 26.59; 0.54)	12/12 (100%; 35.82; 1.08)
1.25 × 10^6^	10^6^	10^5^	500	12/12 (100%; 30.59; 0.46)	12/12 (100%; 32.67; 0.79)
1.25 × 10^5^	10^5^	10^4^	500	12/12 (100%; 34.35; 1.09)	12/12 (100%; 32.97; 0.74)
1.25 × 10^4^	10^4^	10^3^	500	8/12 (66.7%; 37.22; 0.73)	12/12 (100%; 33.73; 0.63)
1.25 × 10^3^	10^3^	10^2^	500	1/12 (8%; 40.71)	12/12 (100%; 32.24; 0.60)
0	0	0	0	0/12	0/12

^a^The dynamic range of the generic PCR was determined on 10-fold dilution series of DENV* in vitro* RNA in the presence of a fixed amount of IC spiked into lysis buffer containing DENV negative human serum as background and for 12 replicates per dilution.

^b^Number of copies cDNA/PCR has been calculated as 1/12.5 of the extracted RNA. assuming 100% efficiency in extraction, reverse transcription (RT), and PCR.

SD: standard deviation.

**Table 4:** LOD of the generic real-time PCR^a^.

DENV* in vitro* RNA copies in PCR^a^	IC copies in PCR^b^	Number positive DENV *in vitro* RNA (%; mean Cq-value: SD)	Number positive IC (%; mean Cq-value: SD)
2500	500	20/20 (100%; 35.98; 0.99)	20/20 (100%; 33.43; 0.65)
1250	500	12/20 (60%; 36.96; 1.13)	20/20 (100%; 32.84; 0.53)
1000	500	10/20 (50%; 37.33; 0.94)	20/20 (100%; 33.66; 0.53)
625	500	8/20 (40%; 38.72; 1.82)	20/20 (100%; 32.61; 0.59)
312	500	5/20 (25%; 39.17; 2.67)	20/20 (100%; 32.69; 0.34)
0	0	0/20	0/20

^a^LOD of the generic PCR was determined by 2-fold dilution series with 20 replicates per dilution of DENV *in vitro* RNA in the presence of a fixed amount of IC spiked into lysis buffer containing DENV negative human serum as background.

^b^number of copies cDNA/PCR has been calculated as 1/12.5 of the extracted RNA, assuming 100% efficiency in extraction, reverse transcription (RT) and PCR.

LOD: lower limit of detection; SD: standard deviation.

**Table 5:** Inter-assay variation of generic real-time PCR^a^.

Run and DENV *in vitro* RNA copies in PCR^a^	IC copies in PCR^b^	Number positive DENV* in vitro* RNA (%; mean Cq-value: SD)	Number positive IC (%; mean Cq-value: SD)	SD between the 3 runs
Run 1				
10^6^	500	4/4 (100%; 26.28; 0.43)	4/4 (100%; 36.21; 1.21)	0.27
10^5^	500	4/4 (100%; 30.40; 0.39)	4/4 (100%; 33.10; 1.37)	0.35
10^4^	500	4/4 (100%; 33.78; 0.38)	4/4 (100%; 32.17; 0.30)	0.22
10^3^	500	2/4 (50%; 36.99; 0.37)	4/4 (100%; 33.64; 0.29)	0.65
10^2^	500	0/4	4/4 (100%; 32.47; 0.20)	—
0	0	0/4	0/4	—

Run 2				
10^6^	500	4/4 (100%; 26.71; 0.56)	4/4 (100%; 36.11; 0.84)	0.27
10^5^	500	4/4 (100%; 30.35; 0.19)	4/4 (100%; 32.66; 0.65)	0.35
10^4^	500	4/4 (100%; 34.31; 0.64)	4/4 (100%; 33.32; 0.39)	0.22
10^3^	500	3/4 (75%; 37.08; 0.86)	4/4 (100%; 33.64; 0.92)	0.65
10^2^	500	1/4 (25%; 37.83)	4/4 (100%; 32.73; 1.09)	—
0	0	0/4	0/4	—

Run 3				
10^6^	500	4/4 (100%; 26.79; 0.60)	4/4 (100%; 35.13; 1.06)	0.27
10^5^	500	4/4 (100%; 30.98; 0.50)	4/4 (100%; 32.47; 0.10)	0.35
10^4^	500	4/4 (100%; 34.27; 0.78)	4/4 (100%; 33.24; 0.54)	0.22
10^3^	500	1/4 (25%; 38.17)	4/4 (100%; 33.91; 0.67)	0.65
10^2^	500	0/4	4/4 (100%; 32.59; 0.23)	—
0	0	0/4	0/4	—

^a^The inter-assay variation was determined with 10-fold dilution series of DENV* in vitro* RNA in the presence of a fixed amount of IC spiked into lysis buffer containing DENV negative human serum as background and for 4 replicates per each dilution. The variation was determined in three separate runs performed at three different days.

^b^Number of copies cDNA/PCR has been calculated as 1/12.5 of the extracted RNA, assuming 100% efficiency in extraction, reverse transcription (RT), and PCR.

SD: standard deviation.

**Table 6:** Summary of patient characteristics and sensitivity and specificity of PCR assays.

Patient characteristics and test parameters	Vietnamese patients with DENV		Ill-returned travelers with DENV		Other causes of fever in travelers	Patients positive for	
	Primary infection	Secondary infection	Primary infection	Secondary infection	OFI	HCV/HAV	Total
Patients (*n*)	5	67	21	16	9	(18)^d^	136
Age; median (range)	10.9 (6.6–12.8)	18.9 (5.6–53.1)	38.7 (17.4–62.6)	33.2 (24.6–73.6)	39.5 (26.3–64.0)	unknown	26.3 (6.6–73.6)
Sex (M/F)	3/2	48/19	9/12	6/10	6/3	unknown	72/46
Days of illness at presentation; median (range)	1 (1-1)	1 (1–4)	6 (1–12)	8.5 (4–22)	—	—	2 (1–22)
Days of DENV viremia; median (range)	1 (1-1)	1 (1–4)	4 (1–11)	7 (4–9)	—	—	1 (1–11)
Patients (*n*) with a positive result in							
Type-specific PCR^a^	5	66	16	9	0	ND	96
Generic PCR^b^	3	66	15	8	0	0	92
Multiplex PCR^c^	3	63	11	3	0	ND	80
Assay characteristics of Generic PCR							
Sensitivity; % (95% CI)	60.0 (23.1–88.2)	100.0 (94.5–100.0)	93.8 (71.7–99.0)	88.9 (56.5–98.0)	—	—	95.8 (89.8–98.4)
*κ*-value	0.736	1.000	0.944	0.924			0.846
Specificity; %					100.0	100.0	100.0

^a^The results were earlier generated by the type-specific primer PCR according to according Laue et al. [11, 16, 17].

^b^Internally controlled generic real-time PCR for DENV.

^c^Multiplex PCR with type-specific probes was only done on samples with a positive generic PCR^b^result.

^d^16 patients were antibody and PCR positive for HCV and 2 patients were antibody and PCR positive for HAV.

OFI: other febrile illness; HCV: hepatitis C virus; HAV: hepatitis A virus; M: male; F: female; 95% CI: 95% confidence interval, ND: not done.

**Table 7:** Serotyping by multiplex real-time PCR with serotype-specific probes.

Serotype	No. of patients positive in the following assays			No. of infections by two serotypes detected by the following assays (DENV serotypes in infections)^c^	
	Type-specific PCR^a^	Generic PCR	Multiplex PCR^b^	Type-specific PCR^a^	Multiplex PCR^b^
1	39	Pos (39)	30	3 (1 + 2)	4 (1 + 4)
2	19	Pos (19)	17	0	1 (2 + 1)
3	3	Pos (3)	2	0	1 (3 + 4)
4	31	Pos (31)	31	0	1 (4 + 2)
total	92	92	80	3	7

Pos: generic PCR can make no distinction between the serotypes.

^a^The results were earlier generated by the type-specific primer PCR according to Laue et al. [11, 16, 17].

^b^Multiplex real-time PCR with serotype-specific probes was only done on positive generic PCR results.

^c^A DENV infection by two serotypes has been assigned to the serotype which signal came up first and showed the highest viral load of the two.

## Abbreviations

DENV: Dengue virus
IC: Internal control
HCV: Hepatitis C
HAV: Hepatitis A
PCR: Polymerase chain reaction
RT: Reverse transcription
DHF: Dengue hemorrhagic fever
DSS: Dengue shock syndrome
NS1: Nonstructural 1 protein
Ab: Antibody
Ag: Antigen
LOD: Lower limit of detection
Cq-value: Cycle for quantification.

## References

[1] Kroeger A, Nathan MB (2006). Dengue: setting the global research agenda. *The Lancet*.

[2] http://whqlibdoc.who.int/publications/2009/9789241547871_eng.pdf.

[3] Beatty M, Letson W, Edgil D, Margolis H (2007). Estimating the total population at risk for locally acquired dengue infection. *American Society of Tropical Medicine and Hygiene*.

[4] Schwartz E, Weld LH, Wilder-Smith A (2008). Seasonality, annual trends, and characteristics of dengue among Ill returned travelers, 1997–2006. *Emerging Infectious Diseases*.

[5] Burke DS, Nisalak A, Johnson DE, Scott McN. R. R (1988). A prospective study of dengue infections in Bangkok. *American Journal of Tropical Medicine and Hygiene*.

[6] Endy TP, Chunsuttiwat S, Nisalak A (2002). Epidemiology of inapparent and symptomatic acute dengue virus infection: a prospective study of primary school children in Kamphaeng Phet, Thailand. *American Journal of Epidemiology*.

[7] Phuong HL, Thai KTD, Nga TTT (2009). Detection of dengue nonstructural 1 (NS1) protein in Vietnamese patients with fever. *Diagnostic Microbiology and Infectious Disease*.

[8] Leparc-Goffart I, Baragatti M, Temmam S (2009). Development and validation of real-time one-step reverse transcription-PCR for the detection and typing of dengue viruses. *Journal of Clinical Virology*.

[9] Fink J, Gu F, Vasudevan SG (2006). Role of T cells, cytokines and antibody in dengue fever and dengue haemorrhagic fever. *Reviews in Medical Virology*.

[10] Chien LJ, Liao TL, Shu PY, Huang JH, Gubler DJ, Chang GJJ (2006). Development of real-time reverse transcriptase PCR assays to detect and serotype dengue viruses. *Journal of Clinical Microbiology*.

[11] Laue T, Emmerich P, Schmitz H (1999). Detection of dengue virus RNA in patients after primary or secondary dengue infection by using the TaqMan automated amplification system. *Journal of Clinical Microbiology*.

[12] Beld M, Minnaar R, Weel J (2004). Highly sensitive assay for detection of enterovirus in clinical specimens by reverse transcription-PCR with an armored RNA internal control. *Journal of Clinical Microbiology*.

[13] Benschop K, Molenkamp R, van der Ham A, Wolthers K, Beld M (2008). Rapid detection of human parechoviruses in clinical samples by real-time PCR. *Journal of Clinical Virology*.

[14] Boom R, Sol CJA, Salimans MMM, Jansen CL, Wertheim-Van Dillen PME, Van Der Noordaa J (1990). Rapid and simple method for purification of nucleic acids. *Journal of Clinical Microbiology*.

[15] Tran TNT, de Vries PJ, Hoang LP (2006). Enzyme-linked immunoassay for dengue virus IgM and IgG antibodies in serum and filter paper blood. *BMC Infectious Diseases*.

[16] Thai KTD, Wolthers KC, van Vugt M, de Vries PJ (2009). Dengue fever among ill returned travelers and concurrent infection by two dengue virus serotypes. *Dengue Bulletin*.

[17] Thai KTD, Phuong HL, Thanh Nga TT (2010). Clinical, epidemiological and virological features of dengue virus infections in vietnamese patients presenting to primary care facilities with acute undifferentiated fever. *Journal of Infection*.

[18] Naze F, Le Roux K, Schuffenecker I (2009). Simultaneous detection and quantitation of Chikungunya, Dengue and West Nile viruses by multiplex RT-PCR assays and Dengue virus typing using High Resolution Melting. *Journal of Virological Methods*.

